# Predicting GPR40 Agonists with A Deep Learning‐Based Ensemble Model

**DOI:** 10.1002/open.202300051

**Published:** 2023-07-05

**Authors:** Jiamin Yang, Chen Jiang, Jing Chen, Lu‐Ping Qin, Gang Cheng

**Affiliations:** ^1^ School of Pharmaceutical Sciences Zhejiang Chinese Medical University Hangzhou P. R. China 310053

**Keywords:** agonist, dataset, deep learning, ensemble model, G protein-coupled receptor 40

## Abstract

Recent studies have identified G protein‐coupled receptor 40 (GPR40) as a promising target for treating type 2 diabetes mellitus, and GPR40 agonists have several superior effects over other hypoglycemic drugs, including cardiovascular protection and suppression of glucagon levels. In this study, we constructed an up‐to‐date GPR40 ligand dataset for training models and performed a systematic optimization of the ensemble model, resulting in a powerful ensemble model (ROC AUC: 0.9496) for distinguishing GPR40 agonists and non‐agonists. The ensemble model is divided into three layers, and the optimization process is carried out in each layer. We believe that these results will prove helpful for both the development of GPR40 agonists and ensemble models. All the data and models are available on GitHub. (https://github.com/Jiamin‐Yang/ensemble_model)

## Graphical abstract

Various GPR40 agonists and non‐agonists for model training and evaluation were collection and used for building and systematically optimizing an ensemble model for predicting GPR40 agonists. The ensemble model was built based on 20 baseline models, which were consisting of different algorithms and molecular representations. And the ensemble model showed greater performance than the performance of any baseline model.

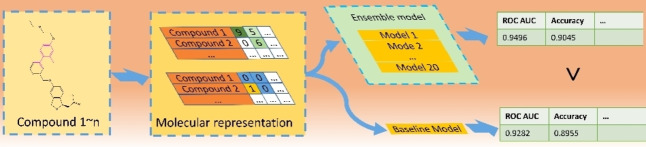

## Introduction

Type 2 diabetes mellitus (T2DM) is a chronic disease characterized by reduced insulin sensitivity, which results in the inability to maintain glucose homeostasis. It is the most prevalent type of diabetes, accounting for over 90 % of all diabetes cases worldwide.^[1]^ Multiple factors, such as genetic architecture and energy‐dense diets, contribute to the development of T2DM.^[2]^ According to statistics, the T2DM epidemic poses a serious threat to global health, affecting 537 million people worldwide.^[1]^ Complications associated with T2DM, including coronary heart disease and cerebrovascular disease, are also alarming.^[3]^ The high incidence of the disease and its serious complications place a tremendous burden on the healthcare system worldwide.

**Figure 1:**
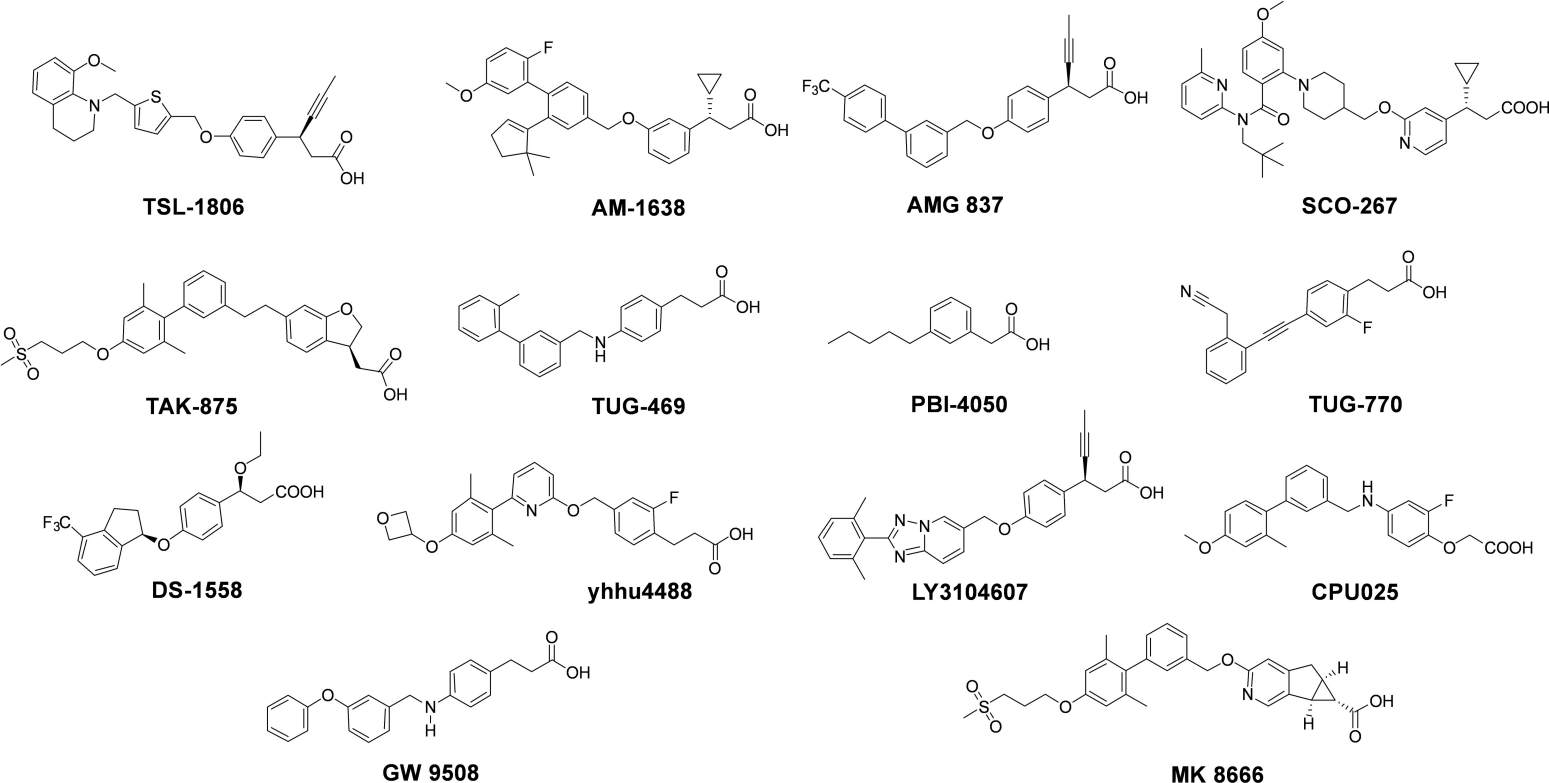
Representative GPR40 agonists.

G protein‐coupled receptor 40 (GPR40), named fatty acid receptor 1 (FFAR1), is a type of G protein‐coupled receptor that is highly expressed in pancreatic β‐cells.^[4]^ It has been shown that GPR40 agonists play an important part in glucose‐stimulated insulin secretion through at least two distinct Gαq‐mediated mechanisms (agonist‐induced IP3 production amplifies glucose‐induced Ca^2+^ oscillations and agonist‐induced protein kinase C (PKC)/(PKD) activation enhances downstream secretory mechanisms that are independent of Ca^2+^ oscillations.), offering a potential new way to treat T2DM.^[5]^ In comparison to current hypoglycemic agents, which often have adverse effects such as hypoglycemia (insulin) or an increased risk of cardiovascular disorders (sulfonylureas), GPR40 agonists stand out due to their low risk of causing hypoglycemia.^[6]^ As shown in Figure 1, numerous small molecule GPR40 agonists have been discovered by researchers and pharmaceutical companies, and several compounds are undergoing clinical trials, such as fezagepras (Liminal BioScience Inc., Canada), TSL‐1806 (Tasly Diyi Pharmaceutical, China), and IDG‐16177 (Ildong Pharmaceutical, Korea).^[7]^ In recent years, additional benefits of GPR40 agonists have been reported, such as cardioprotection and reduced glucagon levels.^[8]^ Therefore, attention should be drawn to the development of GPR40 agonists for treating T2DM.

The development of GPR40 agonists is hindered by both the time‐consuming and labor‐intensive nature of traditional experimental methods and the low selectivity, high toxicity, or low efficiency of certain GPR40 agonists.^[9]^ However, the advent of computer‐aided design drug (CADD) provides an opportunity to increase the efficiency of drug development.^[10]^ In particular, machine learning has proven to be a valuable tool in CADD, allowing for the construction of accurate models to predict compound properties. For instance, extreme gradient boosting was used to predict histone deacetylase 3 inhibitors using Morgan2 fingerprints.^[11]^ Deep learning, belonging to machine learning, has also gained considerable attention for its potential in drug discovery in recent years. For example, the directed message passing neural network have been used with a transfer learning technique to predict a broad‐spectrum anti‐beta‐coronavirus compound along.^[12]^ Notably, although machine learning is used widely in other fields, it is not used enough for screening GPR40 agonists. Most researchers used models like QSAR,^[13]^ pharmacophore modeling,^[14]^ molecular docking,^[15]^ and molecular dynamics^[16]^ instead. In comparison to these approaches, deep learning has several advantages, including its ability to utilize multiple data sources, such as activity data and chemical structure information, and handle large‐scale data.^[17]^ Therefore, developing GPR40 agonist prediction models based on deep learning or machine learning has certain prospects.

Ensemble learning involves combining multiple models (such as machine learning model and deep learning model) to solve a computational intelligence problem and has emerged as an effective way to improve model performance.^[18]^ Previous studies have demonstrated that the ensemble models show improved performance compared to their single baseline models.^[19]^ Moreover, expanding the dataset used for training may improve the performance of the model. Collaboration between a larger dataset and ensemble models has the potential to improve the creation of more accurate prediction models and accelerate the development of GPR40 agonists.

**Figure 2:**
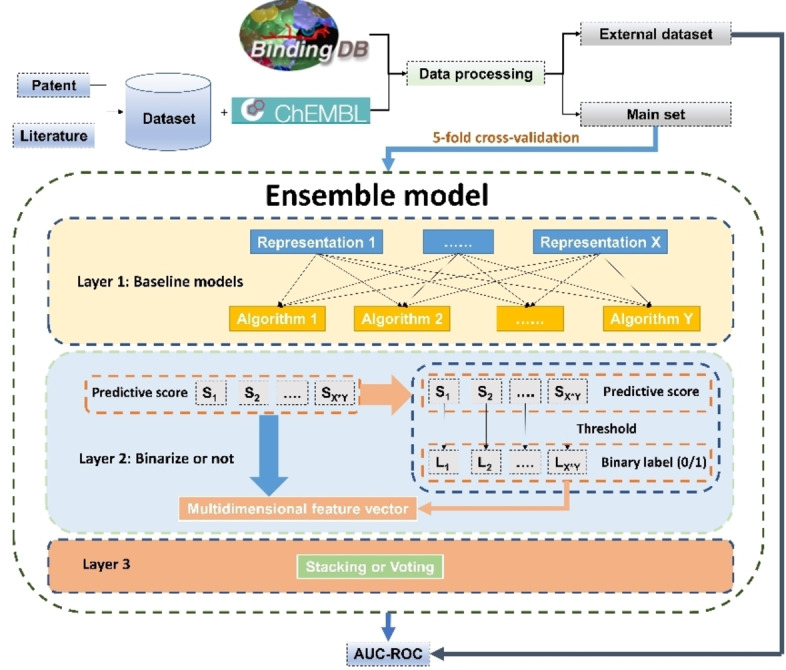
Ensemble model architecture. The ensemble model was comprised of three layers, each corresponding to a distinct process: the construction of baseline models, the conversion of predictive scores to multidimensional feature vectors, and the method for constructing the ensemble model. The data points from ChEMBL, BindingDB, patents, and literature were processed to form two datasets: the main set and the external dataset. Using the main set, the ensemble model was developed through 5‐fold cross‐validation. The external set was then utilized to evaluate the performance of the ensemble model. Algorithm 1, 2, 3, 4, 5 and 6 stand for XGBoost, logistic regression, random forest, support vector machine, fully connected neural network and directed message passing neural network.

**Figure 3:**
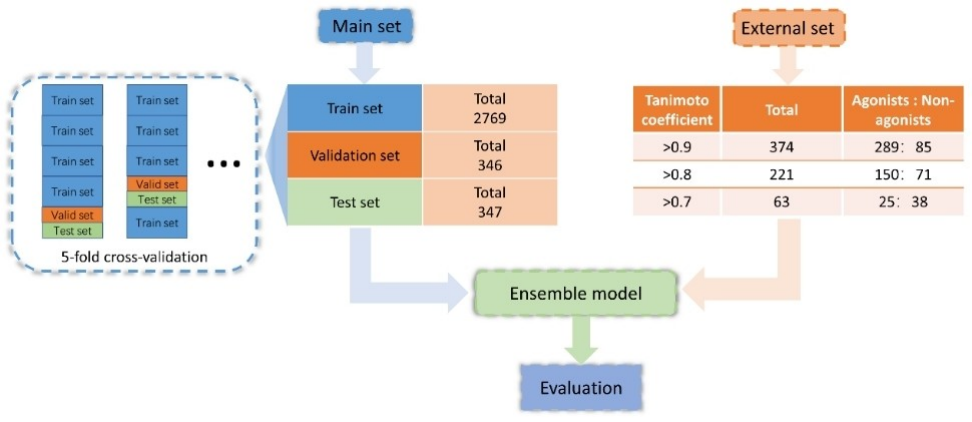
Direction of data flow.

This study describes a novel predictive ensemble model combining ML and DL for identifying GPR40 agonists and non‐agonists. Initially, GPR40 data points were collected from ChEMBL^[20]^ and BindingDB^[21]^ as well as patents and scientific articles. We then established 151 baseline models using these data points and used a stacking strategy to construct the ensemble model (the workflow was outlined in Figure 2 and the data flow is schematically detailed in Figure 3). After systematic optimization of the ensemble model using ROC AUC (the area under the receiver operating characteristic curve) as the evaluation metric, we found that the final ensemble model achieved significantly better performance (ROC AUC: 0.9496) than any baseline models on the external dataset.

## Results and Discussion

### Performance of baseline models with different algorithms and molecular representations

Initially, we built 151 baseline models using 6 different algorithms and 25 molecular representations. The D‐MPNN algorithm (directed message passing neural network) can be trained solely based on the input of the compound's SMILES, without relying on additional molecular representations as input. These models were evaluated on both the test set and the external dataset. The performance of top 20 baseline models in 5‐fold cross‐validation are shown in Table 1, while their performance in additional metrics (such as accuracy, F1, MCC etc.) can be found in Supplementary Tables 4 and 5. The best performing model was found to be based on the FCNN algorithm (fully connected neural network) and AtomPairFP fingerprint, with an ROC AUC of 0.9282 on the external dataset. Secondly, the D‐MPNN algorithm was found to have relatively good performance as it appeared frequently among the top 20 baseline models. Additionally, models based on the AtomPairFP fingerprint also demonstrated good performance. Finally, as the number of removed compounds increased, the predictive abilities of the baseline models decreased.

**Table 1:** Performance of top 20 baseline models.

Algorithm	Representation	ROC_AUC				
		Test^[b]^	External^[b]^			
			All^[c]^	sim<0.9^[c]^	sim<0.8^[c]^	sim<0.7^[c]^
**FCNN**	**AtomPairFP**	0.9417±0.0246	**0.9282**±0.0097	**0.9204**±0.0092	**0.8834**±0.0186	**0.8352**±0.0238
RF	Autocorr	0.9225±0.0240	0.9274±0.0090	0.9198±0.0093	0.8744±0.0166	**0.8652**±0.0234
D‐MPNN	InfoContent	0.9423±0.0207	0.9155±0.0112	0.9069±0.0136	0.8579±0.0207	0.8076±0.0764
D‐MPNN	Property	0.9442±0.0240	0.9099±0.0102	0.8988±0.0138	0.8454±0.0300	0.7745±0.0591
Logreg	AtomPairFP	0.9287±0.0251	0.9050±0.0206	0.8960±0.0204	0.8533±0.0338	0.8194±0.0668
D‐MPNN	Topology	0.9375±0.0260	0.9045±0.0064	0.8943±0.0072	0.8447±0.0081	0.8011±0.0427
XGB	AtomPairFP	0.9442±0.0184	0.9024±0.0109	0.8891±0.0101	0.8245±0.0208	0.6581±0.0308
D‐MPNN	AtomPairFP	0.9355±0.0228	0.9019±0.0201	0.8903±0.0214	0.8260±0.0311	0.7907±0.0586
D‐MPNN	Kappa	0.9350±0.0256	0.8994±0.0133	0.8874±0.0140	0.8331±0.0143	0.7507±0.0308
RF	AtomPairFP	**0.9471**±0.0179	0.8951±0.0070	0.8811±0.0050	0.7770±0.0108	0.7058±0.0112
D‐MPNN	Matrix	0.9369±0.0263	0.8916±0.0283	0.8765±0.0336	0.7999±0.0532	0.7293±0.1144
D‐MPNN	Connectivity	0.9400±0.0232	0.8909±0.0256	0.8807±0.0310	0.8119±0.0540	0.7006±0.1476
FCNN	MAP4	0.9393±0.0251	0.8908±0.0132	0.8714±0.0139	0.7821±0.0258	0.7865±0.0271
D‐MPNN	Path	0.9343±0.0242	0.8874±0.0382	0.8729±0.0455	0.8093±0.0720	0.7324±0.1634
RF	MorganFP	0.9420±0.0223	0.8868±0.0032	0.8667±0.0037	0.7553±0.0073	0.5356±0.0105
D‐MPNN	N.A.^[a]^	0.9309±0.0284	0.8801±0.0177	0.8710±0.0212	0.8263±0.0282	0.8448±0.0417
RF	MAP4	0.9389±0.0245	0.8795±0.0088	0.8586±0.0102	0.7405±0.0170	0.6712±0.0371
XGB	MAP4	0.9416±0.0244	0.8782±0.0150	0.8590±0.0172	0.7621±0.0301	0.6714±0.0496
SVM	AtomPairFP	0.9051±0.0221	0.8747±0.0248	0.8684±0.0271	0.8153±0.0356	0.8135±0.0926
D‐MPNN	PharmacoErGFP	0.9335±0.0262	0.8713±0.0075	0.8559±0.0080	0.7663±0.0063	0.8194±0.0150

[a] The D‐MPNN was trained without additional molecular representations as input. [b] Evaluating the ensemble model based on a test set or external dataset. [c] according to different Tanimoto similarity (0.9, 0.8 and 0.7), removing compounds in the external dataset with respect to the main set molecules. Standard deviation=sqrt(∑(a_i−b) 2/(n−1)), a_i: each value in the data set, n: the number of data points, and b: the average value of the data set. The SD calculation methods below are the same. All models were developed using 5‐fold cross‐validation method.

### Performance of ensemble models with different numbers of nodes in the hidden layer of layer 3

As illustrated in Supplementary Tables 6 and 7, we investigated the impact of the optimizer and size of hidden layers on the performance of FCNN and AtomPairFP was utilized as a molecular representation to provide compound information for the training and evaluation of the model. Our findings suggest that the ensemble models consisting of a single hidden layer with 800 nodes and using the Adam optimizer demonstrated improved performance. To verify the parameters of FCNN in the ensemble model further, the number of nodes in the hidden layer of layer 3 was optimized. Table 2 demonstrates that the number of nodes was set to 200, 400, 600, 800, 1000 and 1200. With 151 top baseline models selected, all were utilized to construct the ensemble model. The comparison of the ROC AUC between the ensemble models on the test set and external dataset revealed that the best overall performance was achieved with 800 nodes. As a result, we selected 800 nodes for further optimization of the ensemble model.

**Table 2:** The optimization of the size of the hidden layer in layer 3 (FCNN algorithm).

No. of baseline models^[a]^	No. of nodes^[b]^	ROC AUC	
		Test^[c]^	External^[c]^
151	200	0.9425±0.0236	0.9249±0.0232
	400	0.9454±0.0219	0.9398±0.0085
	600	0.9445±0.0227	0.9376±0.0097
	800	0.9437±0.0231	0.9401±0.0075
	1000	0.9426±0.0233	0.9381±0.0086
	1200	0.9429±0.0237	0.9405±0.0077

[a] Selected different numbers of top baseline models to construct the layer 1 of the ensemble model. [b] Number of nodes in the hidden layer of FCNN. [c] Evaluating the ensemble model based on a test set or external dataset. All models were developed using 5‐fold cross‐validation method.

### Performance of ensemble models with different numbers of top baseline models in layer 1

Here, we investigated the impact of the number of top baseline models in layer 1 on the performance of the ensemble model. Table 3 shows that the ROC AUC of the ensemble model on the external dataset initially decreased as the number of top baseline models decreased, but then increased until the number of top baseline models was 20. Based on the ROC AUC of the ensemble model on the test set, we observed that the performance fluctuated only slightly when the number of top baseline models was lower than 70. To summarize, we determined that 20 is the optimal number of top baseline models for further experiments.

**Table 3:** The process of optimizing the ensemble model with different numbers of top baseline models in layer 1.

No. of baseline models^[a]^	ROC_AUC	
	Test^[b]^	External^[b]^
140	0.9425±0.0245	0.9350±0.0072
130	0.9422±0.0242	0.9398±0.0064
120	0.9428±0.0235	0.9343±0.0059
100	0.9420±0.0230	0.9346±0.0067
90	0.9436±0.0210	0.9213±0.0068
80	0.9435±0.0201	0.9191±0.0050
70	0.9481±0.0206	0.9259±0.0058
60	0.9531±0.0159	0.9295±0.0056
50	0.9501±0.0192	0.9274±0.0041
40	0.9557±0.0166	0.9382±0.0051
30	0.9538±0.0171	0.9417±0.0055
25	0.9531±0.0173	0.9457±0.0063
20	0.9524±0.0172	**0.9496**±0.0057
15	0.9491±0.0161	0.9475±0.0122
10	0.9481±0.0158	0.9410±0.0149

[a] Selected different numbers of top baseline models to construct the layer 1 of the ensemble model. This section used 800 nodes in the hidden layer of FCNN. [b] Evaluating the ensemble model based on a test set or external dataset. All models were developed using 5‐fold cross‐validation method.

### The performance of ensemble models based on different layer 2

In layer 2, the impact of binarizing the predictive scores of the baseline models on the ensemble model by setting various thresholds (0.3, 0.4, 0.5, 0.6, and 0.7) for binarization was investigated. As shown in Table 4, the best ROC AUC of the ensemble model based on binarization was 0.9358 on the external dataset and 0.9319 on the test set, with a threshold of 0.5 being the optimal threshold for binarization. However, the ROC AUC was even higher without binarization, achieving 0.9496 on the external dataset and 0.9524 on the test set. As a result, we decided not to binarize the predictive scores in layer 2 and continue with the rest of the experiment.

**Table 4:** The performance of ensemble models based on different layer 2. Here, the FCNN utilizes a single hidden layer of 800 nodes, and the top 20 baseline models are used to construct the ensemble model.

Whether the probability is binarized in layer 2	Threshold	ROC AUC	
		Test^[a]^	External^[a]^
binary label	0.3	0.9318±0.0240	0.9352±0.0082
	0.4	0.9314±0.0240	0.9356±0.0095
	0.5	0.9319±0.0244	0.9358±0.0089
	0.6	0.9316±0.0238	0.9357±0.0086
	0.7	0.9315±0.0241	0.9350±0.0089
**original**	N.A.^[b]^	**0.9524**±0.0172	**0.9496**±0.0057

[a] Evaluating the ensemble model based on a test set or external dataset. [b] Without binarization using different thresholds. All models were developed using 5‐fold cross‐validation method.

### The performance comparison of ensemble models constructed by voting or stacking

The output from layer 2 was processed in layer 3 using two methods: voting and stacking. As shown in Table 5, ensemble models constructed via the voting method improved their performance as the number of top baseline models decreased, with the best ensemble model achieving a ROC AUC of 0.7717 on the external dataset. However, the ROC AUC of the best ensemble model constructed by stacking was superior. Further evaluation on the test set revealed that the performance of the voting method‘s ensemble models no longer significantly improved when the number of top baseline models was reduced below 25. As a result, stacking was chosen as the method for processing data in layer 3.

**Table 5:** The comparison of ensemble models constructed by voting or stacking.

Layer 3	No. of nodes^[a]^	No. of baseline models^[b]^	ROC AUC	
			Test^[c]^	External^[c]^
Voting	N.A.^[d]^	30	0.8376±0.0379	0.7265±0.0387
		25	0.8466±0.0355	0.7343±0.0334
		20	0.8469±0.0356	0.7469±0.0317
		15	0.8476±0.0347	0.7688±0.0292
		10	0.8462±0.0313	0.7717±0.0216
**Stacking**	**800**	**20**	**0.9524**±0.0172	**0.9496**±0.0057

[a] The nodes in the hidden layer of FCNN. [b] Selected different numbers of top baseline models to construct the layer 1 of the ensemble model. [c] Evaluating the ensemble model based on a test set or external dataset. [d] When using voting to build the ensemble model, no neural network hidden layer was used. All models were developed using 5‐fold cross‐validation method.

### Performance of ensemble models evaluated on different external datasets and test set

In this experiment, the number of nodes was set to 800, and various numbers of top baseline models were selected to construct the ensemble model. To identify its performance, we removed compounds from the external dataset based on their similarity to the compounds in the main set. As shown in Table 6, as similar compounds were removed, the performance of the ensemble model started to decrease. After removing compounds with a similarity above 0.7, the performance of the ensemble model even dropped to 0.7 on the external dataset. However, with the number of top baseline models set to 20, ensemble model provided performance with ROC AUC reaching 0.8202 on the external dataset, indicating that it was still a powerful model for distinguishing between GPR40 agonists and non‐agonists.

**Table 6:** The performance of ensemble models evaluated on different external datasets and test set.

No. of baseline models^[a]^	ROC AUC				
	Test^[b]^	External^[b]^			
		all^[c]^	sim<0.9^[c]^	sim<0.8^[c]^	sim<0.7^[c]^
140	0.9425±0.0245	0.9350±0.0072	0.9275±0.0070	0.8669±0.0145	0.7724±0.0369
130	0.9422±0.0242	0.9398±0.0064	0.9336±0.0054	0.8810±0.0113	0.7996±0.0280
120	0.9428±0.0235	0.9343±0.0059	0.9263±0.0037	0.8631±0.0077	0.7672±0.0186
100	0.9420±0.0230	0.9346±0.0067	0.9265±0.0047	0.8648±0.0090	0.7478±0.0221
90	0.9436±0.0210	0.9213±0.0068	0.9099±0.0048	0.8309±0.0106	0.7091±0.0322
80	0.9435±0.0201	0.9191±0.0050	0.9075±0.0046	0.8298±0.0074	0.7358±0.0191
70	0.9481±0.0206	0.9259±0.0058	0.9159±0.0052	0.8477±0.0126	0.7531±0.0352
60	0.9531±0.0159	0.9295±0.0056	0.9198±0.0053	0.8554±0.0104	0.7678±0.0091
50	0.9501±0.0192	0.9274±0.0041	0.9176±0.0053	0.8526±0.0075	0.7665±0.0213
40	0.9557±0.0166	0.9382±0.0051	0.9291±0.0032	0.8716±0.0060	0.8006±0.0075
30	0.9538±0.0171	0.9417±0.0055	0.9333±0.0047	0.8785±0.0086	0.7958±0.0148
25	0.9531±0.0173	0.9457±0.0063	0.9383±0.0057	0.8891±0.0105	0.8103±0.0079
**20**	**0.9524**±0.0172	**0.9496**±0.0057	**0.9429**±0.0057	**0.8999**±0.0103	**0.8202**±0.0066
15	0.9491±0.0161	0.9475±0.0122	0.9407±0.0139	0.8978±0.0233	0.8065±0.0269
10	0.9481±0.0158	0.9410±0.0149	0.9338±0.0176	0.8876±0.0302	0.7878±0.0543

[a] Selected different numbers of top baseline models to construct the layer 1 of the ensemble model This section used 800 nodes in the hidden layer of FCNN. [b] Evaluating the ensemble model based on a test set or external dataset. [c] According to different Tanimoto similarity (0.9, 0.8 and 0.7), removing compounds in the external dataset with respect to the main set molecules. All models were developed using 5‐fold cross‐validation method.

### The performance comparison of the ensemble model and molecular docking

**Figure 4:**
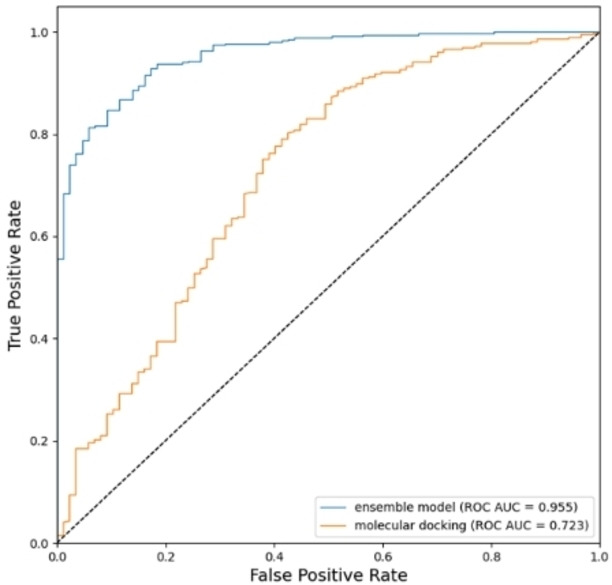
Performance comparison between ensemble model and molecular docking.

In this study, we also performed molecular docking by AutoDock Vina software and evaluated its performance in terms of ROC AUC.^[22]^ The ROC AUC curve was generated based on the docking score and compared to the ROC AUC curve of our ensemble model. As shown in Figure 4, the ROC AUC from molecular docking was inferior to that of our ensemble model. This poor performance by docking is likely due to the high dynamic nature of the GPR40 binding pockets, making it challenging to accurately determine the binding sites for docking ligands.^[23]^

The GPR40 receptor contains at least three allosteric binding sites, and two of them (intrahelical site and extrahelical site) have the potential to be developed as drugs to treat T2DM, as reported.^[24]^ The better docking score was selected between the two different binding site dockings to generate the ROC AUC curve, which demonstrated a stronger binding capability between the ligand and the GPR40 receptor. The protein‐ligand complexes used for molecular docking in this study were obtained from the Protein Data Bank (PDB IDs: 4PHU and 5 KW2).[^23a^, ^23c^] The structures were pre‐processed using AutoDock Tools, removing the solvent and original ligands while preserving the protein‘s structure. The preprocessing of ligands was carried with ChemBio 3D software. These ligands went through the process of energy minimization with an added hydrogen and were assigned a Gasteiger charge. The docking boxes for the receptors in 4PHU and 5 KW2 were generated with a center of −50.59 Å×−1.6 Å×59.6 Å (x×y×z) and 19.22 Å×32.91 Å×29.96 Å (x×y×z) respectively, and both were created within a 20 Å range. Other docking parameters selected the default setting.

### Explanation of the ensemble model with Shapley Additive exPlanations

**Figure 5:**
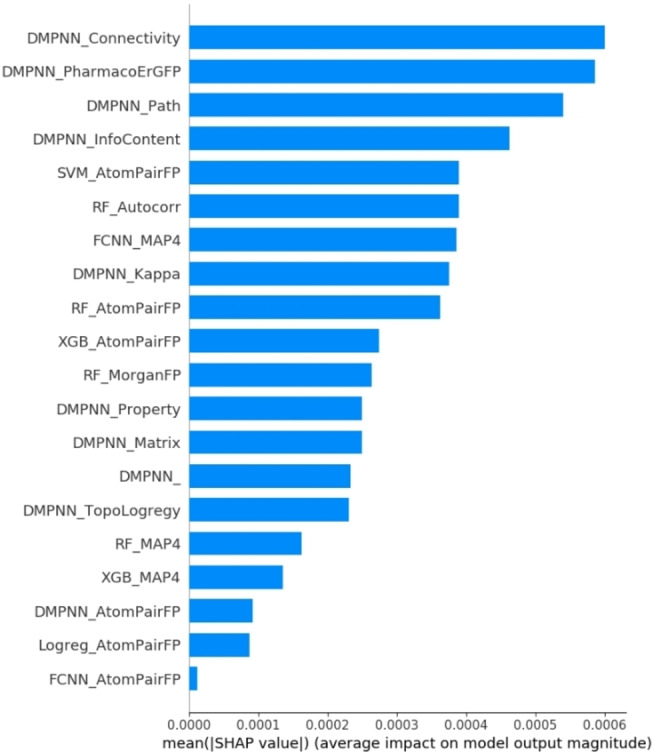
SHAP values of the 20 baseline models.

To explain the output of the ensemble model, we apply the Shapley Additive exPlanations (SHAP)^[25]^ to investigate the influence of each baseline model on the ensemble model. As shown in Figure 5, the twenty baseline models were listed from top to bottom according to their impact on the ensemble model. The SHAP value represented the influence of a baseline model on the ensemble model. The larger the SHAP value, the greater the impact on the ensemble model. In Figure 5, out of the top 20 models ranked by SHAP values, the DMPNN algorithm had a higher frequency of appearing with 10 appearances, surpassing other algorithms. In terms of molecular characterization, the AtomPairFP molecular fingerprint had a higher frequency of appearing with 6 appearances in the top 20 models ranked by SHAP values, surpassing other molecular representations.

## Computational Methods

### Data preparation

Firstly, we extracted two datasets from two public databases (ChEMBL^[20]^ and BindingDB^[21]^). To keep the dataset up‐to‐date, we manually gathered a dataset of 2687 compounds from scientific articles and patents. This dataset included information such as journal name, Simplified Molecular Input Line Entry System (SMILES), and activity among others. Next, we converted the SMILES of all compounds to canonical SMILES by OpenBabel 2.3.1 and it generated a sole string for any particular molecule.^[26]^ Then, binary labels (1/0) were assigned to the compounds, with 1 representing agonists and 0 representing non‐agonists. Considering the ratio of negative samples to positive samples and the EC_50_ value of existing active compounds (TAK‐875: 0.014 μM, SCO‐267: 0.012 μM and so on), the threshold for distinguishing agonists from non‐agonists was determined to be 1 μM and the further details about their measurements can be found in paragraph 1 (supporting information).[^7e^, ^7h^] Fourthly, we combined the manually collected dataset with the dataset from ChEMBL to form the main set, and used the dataset from BindingDB as an external dataset. After that, we checked the SMILES, removed duplicative SMILES, duplicative entities with conflicting labels, salts, and standardized the molecules using MolVS.^[27]^ After removing all of the problematic molecules, the main set consists of 3462 molecules, including 2703 agonists and 759 non‐agonists. The external dataset has 440 molecules, with 353 agonists and 87 non‐agonists. As shown in Supplementary Figures 4, 5 and 6, we conducted principal component analysis three times, which was based on AtomPairFP, or MorganFP, or six molecular properties including the molecular weight, topological polar surface area, number of rotatable bonds, number of hydrogen bond donors, number of hydrogen bond acceptors and logP. The molecules in the external dataset are mostly distributed within the space of the main set, which indicates that the external set is appropriate for evaluating the performance of the model.

### Algorithms and molecular representation calculation

A baseline model consists of an algorithm and a molecular representation. While ML continues to advance rapidly, traditional ML algorithms may struggle to achieve desirable results when handling imbalanced, high‐dimensional data. On the other hand, DL can extract abstract features from a training set and effectively process complex and heterogeneous data structures.^[28]^ In this work, both ML algorithms (XGBoost, logistic regression, random forest, and support vector machine) and DL algorithms (fully connected neural network, directed message passing neural network) were chosen for exploring and building models. The algorithms were built using sklearn,^[29]^ keras,^[30]^ xgboost^[31]^ and Chemprop,^[32]^ and their parameters were based on those of DeepChem^[33]^ as shown in Supplementary Tables 1 and 2. The principles of these algorithms were briefly discussed in the following paragraphs. Next, we calculated the molecular fingerprints and molecular descriptors by RDkit,^[34]^ MolMap^[35]^ and mordred.^[36]^ In this article, twelve kinds of molecular fingerprints (MorganFP, RDkitFP, AtomPairFP, TorsionFP, AvalonFP, EstateFP, MACCSFP, PharmacoErGFP, PharmacoPFP, PubChemFP, MHFP6 and MAP4) and thirteen molecular descriptors (MOE, Property, Constitution, Autocorr, Fragment, Charge, Estate, Connectivity, Topology, Kappa, Path, Matrix, InfoContent) were calculated. The information and sources of these 25 molecular representations can be found in Supplementary Table 3. Additionally, our analysis evaluated the impact of chiral fingerprints on overall model performance and found that it had little effect. See Supplementary Table 5 for detailed results. In short, the aim was to develop a powerful ensemble model based on these algorithms and molecular representations.

### XGBoost

The XGBoost (XGB) algorithm is widely utilized in various fields and provides state‐of‐the‐art results on multiple problems.^[31]^ It can handle both classification and regression problems by selecting different baseline models, and the final result is achieved by adding new models to the existing model until its performance is no longer greatly improved. The main advantage of XGB is its speed, thanks to its parallel and cache‐aware computing.^[37]^ Additionally, it includes a regularization term to prevent over‐fitting and shrinkage and column subsampling to further reduce the risk of over‐fitting. Beyond that, XGB can deal with the sparsity pattern (e. g. missing values) in the data, which is significant for a model to handle real‐world data. XGB also has the advantage that it implements out‐of‐core computation with block compression and block sharding to achieve scalable learning and could be used to deal with billions of samples with just a limited amount of computing resources. Given its excellent performance, we included it in building models for this study.^[38]^

### Logistic regression

Logistic Regression (LR) is a widely used machine learning method with a long history of development.^[39]^ It has been shown in a previous systematic review to be effective for clinical prediction and can also be used for predicting molecular properties, mitochondrial toxicity.^[40]^ Actually, LR is a classification model commonly used for dichotomous problems. First, LR assumes that the logarithm of the odds with respect to the independent variables is linear and the probability distribution of the data is Bernoulli.^[41]^ Next, this algorithm uses maximum likelihood estimation to find a proper parameter set of the model for the calculation of the loss function. Finally, it pursues the minimum loss function for getting a good decision boundary that separates the two types of samples. Besides, there are two methods (Newton‐Raphson method and gradient descent) to calculate the model parameter with which the model's loss function is minimum. There are also other features of LR, such as introducing regularization and parallel computing. Generally, the performance of LR is largely determined by whether the data follows the assumed model. In addition to these fundamentals, It is considered a powerful model for handling low‐dimensional data in binary classification, and was used as one of the algorithms in our baseline models.^[42]^

### Random forest

A single decision tree usually can't provide a good performance in prediction and combining the results of multiple models may yield better result. Random forest (RF) is a traditional machine learning algorithm which is composed of a collection of decision trees. And RF uses a special bagging strategy to construct this ensemble model, which is characterized by selecting a subset of features to build decision trees. Because of this feature randomness, the decision trees of RF have low correlation in their compositions. The assembly of the decision trees also minimizes their individual errors. In terms of applications, RF is always used for classification, regression, and feature selection. For example, Cano et al. discussed the approach based on RF to improve the performance of selecting molecular descriptors.^[43]^ Besides, classification tasks and regression tasks handle the results of decision trees differently. RF averages the prediction scores of the decision trees to obtain the result of the regression task, while a majority vote is used to summarize the results of the decision trees for a classification task.

### Support vector machine

Support vector machine (SVM) algorithm has a long history of development which was created in 1963 and developed rapidly in 1990s. In the case of binary classification problem, SVM searches for a hyperplane which is able to separate two classes with different labels. But there are a lot of hyperplanes that can finish this job and it chooses the best hyperplane forming the largest separation between the different classes.^[44]^ It also maximizes the margin between the best hyperplane and the nearest data point on either side. There are two kinds of SVM (including linear and nonlinear SVM). In linear SVM, soft‐margin is sometimes introduced to avoid the noise of samples and model overfitting. The nonlinear SVM uses kernel trick to deal with the problem that the set is not linearly separable in the original space. In short, the set is mapped into a higher‐dimensional space which makes the set separable linearly. Here, kernel SVM is used as the experiment algorithm.

### Fully connected neural network

With comparison to other DL algorithms, fully connected neural network (FCNN) has the simplest structure and the longest history. Compared with ML models, it has a better ability to process high‐dimensional feature data.^[45]^ In fact, FCNN consists of three kinds of Layers (input Layer, hidden layer and output Layer), and the number of the hidden layer depends on the real needs. Particularly, the nodes which are distributed in two Layers are connected to each other and that constitutes the basic structure of FCNN. The complexity of the model depends on how many hidden Layers are in the model and how many nodes are in each Layer.^[46]^ When the structure of the model is complex, such networks can be hard to be understood for people. For adjusting parameters in the model, there is a process of backpropagation that combines with gradient descent. Furthermore, the performance of FCNN hinges on various hyperparameters, including the number of iterations of gradient descent, the learning rate, the number of hidden Layers and so on. And a study says that it is difficult to fit the samples and easier to overfitting for FCNN with numerous hyperparameters in training.^[47]^ Here, we chose it as the base algorithm because of its important status in DeepChem and we used the default hyperparameters in DeepChem2.1.

### Directed message passing neural network

Directed message passing neural network (D‐MPNN), developed in 2019, has become an effective tool for predicting molecular properties. Although D‐MPNN is similar to the message passing neural network, the main difference is that D‐MPNN employs messages related to directed bonds instead of atoms.^[48]^ Simply, there are two phases of the operation of D‐MPNN: a message passing phase and a readout phase. In phase one, D‐MPNN builds a neural representation of the molecule and the workflow is summarized by the following steps: (1) initialize bond hidden states; (2) choose message passing functions and edge update functions to update bond features; (3) calculate an atom representation of the molecule with bond features. In another phase, D‐MPNN reads the final representation of the molecule, calculates a feature vector for the compound and makes predictions using a feed‐forward neural network. In fact, Yang et al. have described the D‐MPNN exhaustively and shown that using public and proprietary datasets, D‐MPNN consistently matches or outperforms some models that use fixed molecular descriptors or other graph neural architectures.^[32]^ In addition, an open‐source package Chemprop is provided by Yang et al. for building D‐MPNN, in which we apply its classification task. The algorithm's high performance in many areas attracted our attention and we chose it as one of the basic algorithms.

### Construction and Optimization of the Ensemble Model

In this study, 151 baseline models were constructed by combining an algorithm and a molecular representation. The stacking strategy or voting was employed to build ensemble models based on varying numbers of these baseline models. As illustrated in Figure 3, the models were all developed using a 5‐fold cross‐validation method. Both 5‐fold and 10‐fold cross‐validation offers a certain degree of reliability, while using 10‐fold cross‐validation can significantly increase the time required for ensemble model's training and evaluation. In 5‐fold cross‐validation, the main dataset was divided into 5 segments, with 4 segments serving as the training set and the remaining 1 was further split evenly as the validation and test sets. This split process was automatically done by running Chemprop (i. e., D‐MPNN), and other models were trained and evaluated using the identical datasets and 5‐fold splits. And the ensemble model was composed of three layers, namely layer 1, layer 2, and layer 3, as illustrated in Figure 2. As mentioned above, the algorithm and the molecular representation constituted the baseline model in layer 1. In layer 2, there are two approaches to processing the data: keeping the predictive scores as they were, or binarizing them. When binarizing the scores, they were transformed into binary labels (1 or 0) based on a threshold of 0.3, 0.4, 0.5, 0.6, or 0.7. Predictive scores above the threshold were designated as 1, while scores below were designated as 0. It was worth noting that the output labels or the original predictive scores were combined into a multidimensional feature vector which served as input of layer 3. And there were two ways to process this input data in layer 3 to obtain the final result: through an FCNN model or through voting. Next, we explored several aspects to optimize the ensemble model. Firstly, we investigated the impact of the size of the hidden layer in the FCNN on the performance of the ensemble model, and sought the appropriate number of nodes to build the FCNN of layer 3. Secondly, we optimized the ensemble model by adjusting the number of top baseline models in layer 1, as weak classifiers among the baseline models may decrease the ensemble model‘s predictive power. Thirdly, we explored the effect of binarizing the predictive scores. Once we found a stacking ensemble model with good performance, we employed a voting strategy in layer 3 to construct voting ensemble models. And we compared the two types of ensemble models, stacking and voting, to determine which was better. Moreover, the performance of the ensemble model was also evaluated using different external datasets, by excluding compounds with a Tanimoto similarity of 0.9, 0.8, or 0.7 to the main set molecules. The number of compounds in each external dataset is presented in Table 7.

**Table 7:** The number of compounds in different external datasets and main set.

Dataset	Similarity	No. of compounds^[a]^
external dataset	all^[b]^	440
	smi<0.9^[b]^	374
	smi<0.8^[b]^	221
	smi<0.7^[b]^	64
main set		3462

[a] The number of compounds. [b] sim<0.9, sim<0.8, sim<0.7: according to different Tanimoto similarity (0.9, 0.8 and 0.7), removing compounds in the external dataset with respect to the main set molecules.

### Performance evaluation

In this paper, models’ reliability and predictive power were evaluated in two ways, including 5‐fold cross‐validation and external validation, which is performed using an external validation data set. We employed several performance indicators to quantify the performance of the baseline classifiers and the ensemble classifier, including ROC AUC, precision, F1 score, ACC, and MCC.^[49]^ Thereinto, ROC AUC is the most critical measurement of the model's classification capacity, whose value ranges from 0.5 to 1. When the ROC AUC value gets closer to 1, the prediction of the ability of the model is better. These indicator equations are defined as follows [Equations (1) to 7]:

(1) $$precision=\frac{TP}{TP+FP}\;$$

(2) $$accuracy=\frac{TP}{TP+FN}\;$$

(3) $$MCC=\frac{TP\times TN-FP\times FN}{\sqrt{\left(TP+FP)(TP+FN)(TN+FP)(TN+FN\right)}}\;$$

(4) $$NPV=\frac{TN}{TN+FN}\;$$

(5) $$FPR=\frac{FP}{TN+FP}\;$$

(6) $$recall=\frac{TP}{TP+FN}\;$$

(7) $$F1\;score=\frac{2\times precision\times recall}{precision+recall}\;$$

Where TP (true positive) is the number of the agonists that are correctly predicted, TN (true negatives) represents the number of the non‐agonists that are correctly predicted, FN (false negative) is the number of agonists that are mistakenly predicted as non‐agonists, FP (false positive) is the number of the non‐agonists that are mistakenly predicted as agonists.

## Conclusion

For prediction of GPR40 agonists, we initially collected data records from ChEMBL and BindingDB, totaling 2038 data points, and curated 1689 GPR40 agonists and 175 non‐agonists from patents and literature. Next, we proposed an ensemble model for prediction of GPR40 agonists based on both ML and DL techniques. The architecture of the ensemble model can be classified as 3 layers. After optimizing each layer systematically, the ensemble model was significantly improved with an AUC‐ROC of 0.9496. The optimal architecture for the best ensemble model: using the top 20 baseline models as the models in layer 1, without binarizing probabilities in layer 2, and both a single hidden layer with 800 nodes and the Adam optimizer were used for the FCNN in layer 3. We believe that this work affords an ensemble model capable of accurately predicting GPR40 agonists and will contribute to the development of drugs for the treatment of diabetes.

Our research also has limitations, which need to be addressed in the future. Currently, there are two types of GPR40 agonists that bind to different pockets due to the fact that different pockets of the protein may produce different functions: partial agonists and full agonists.[^24^, ^50^] Thus, it is necessary to differentiate between these two types of agonists. However, there is a lack of information on full agonists, making it difficult to differentiate between the two types of agonists. In the future, we will focus on accumulating information on full agonists, in order to train machine learning or deep learning models that can better distinguish between the two types of agonists and analyze their structures.

## Supporting Information

The Supporting Information provides detailed information on various aspects including the data processing procedure, the GPR40 agonist and non‐agonist frameworks, principal component analysis of the training and external datasets using six molecular properties, AtomPairFP and MorganFP, information and sources of various molecular representations, a comparison of our ensemble model with the top 20 baseline models using various evaluation metrics, the impact of chiral fingerprints on the overall performance of the models, the influence of optimizer and hidden layer structure on FCNN performance, and additional information on the measurement of compound activity. All the data and models are opened via GitHub. (https://github.com/Jiamin‐Yang/ensemble_model)

## Conflict of interest

The authors declare no conflict of interest.

1

## Supporting information

As a service to our authors and readers, this journal provides supporting information supplied by the authors. Such materials are peer reviewed and may be re‐organized for online delivery, but are not copy‐edited or typeset. Technical support issues arising from supporting information (other than missing files) should be addressed to the authors.

Supporting Information

## Data Availability

All the data and models in this study are opened. For more information please check github (https://github.com/Jiamin‐Yang/ensemble_model) and zenodo (DOI 10.5281/zenodo.7641975)
