# Supplementary material for: Predicting GPR40 Agonists with A Deep Learning‐Based Ensemble Model
Source: ChemistryOpen. 2023 Jul 5;12(11):e202300051. doi: 10.1002/open.202300051 (PMC10661831; doi:10.1002/open.202300051)
Supplement: Supplementary file 1 — Supporting Information [file OPEN-12-e202300051-s001.pdf]

ChemistryOpen

Supporting Information

Predicting GPR40 Agonists with A Deep Learning-Based Ensemble Model

Jiamin Yang, Chen Jiang, Jing Chen, Lu-Ping Qin,* and Gang Cheng*

Content: Supplementary **Figure 1** shows the detail of the process of data processing, including converting to canonical SMILES, labeling, merging datasets, checking SMILES, removing duplicative SMILES, removing duplicative entities with conflicting labels, removing salt, and molecule standardization (MolVS). Supplementary **Figure 2** and **3** show the frameworks of GPR40 agonists and non-agonists. When the area of the framework in the picture is bigger, the framework is more important. Supplementary **Figure 4**, **5** and **6** showed the principal component analysis of training set and external dataset using six molecular properties, AtomPairFP and MorganFP. Next, supplementary **Table 1** and **2** including the hyperparameter values of different models. And, supplementary **Table 3** provides information and the sources of various molecular representations. The comparison of performance between our ensemble model and the top 20 baseline models using various evaluation metrics is presented in the Supplementary **Table 4** and **5**. Additionally, we explored the impact of chiral fingerprints on the overall performance of the models and the results can be found in the Supplementary **Table 6**. Supplementary **Table 7** and **8** investigated the influence of optimizer and hidden layer structure on FCNN performance. Our analysis suggested that the FCNN based on a single hidden layer with 800 nodes and the Adam optimizer was the optimal. **Paragraph 1** provides further information on the measurement of compound activity.

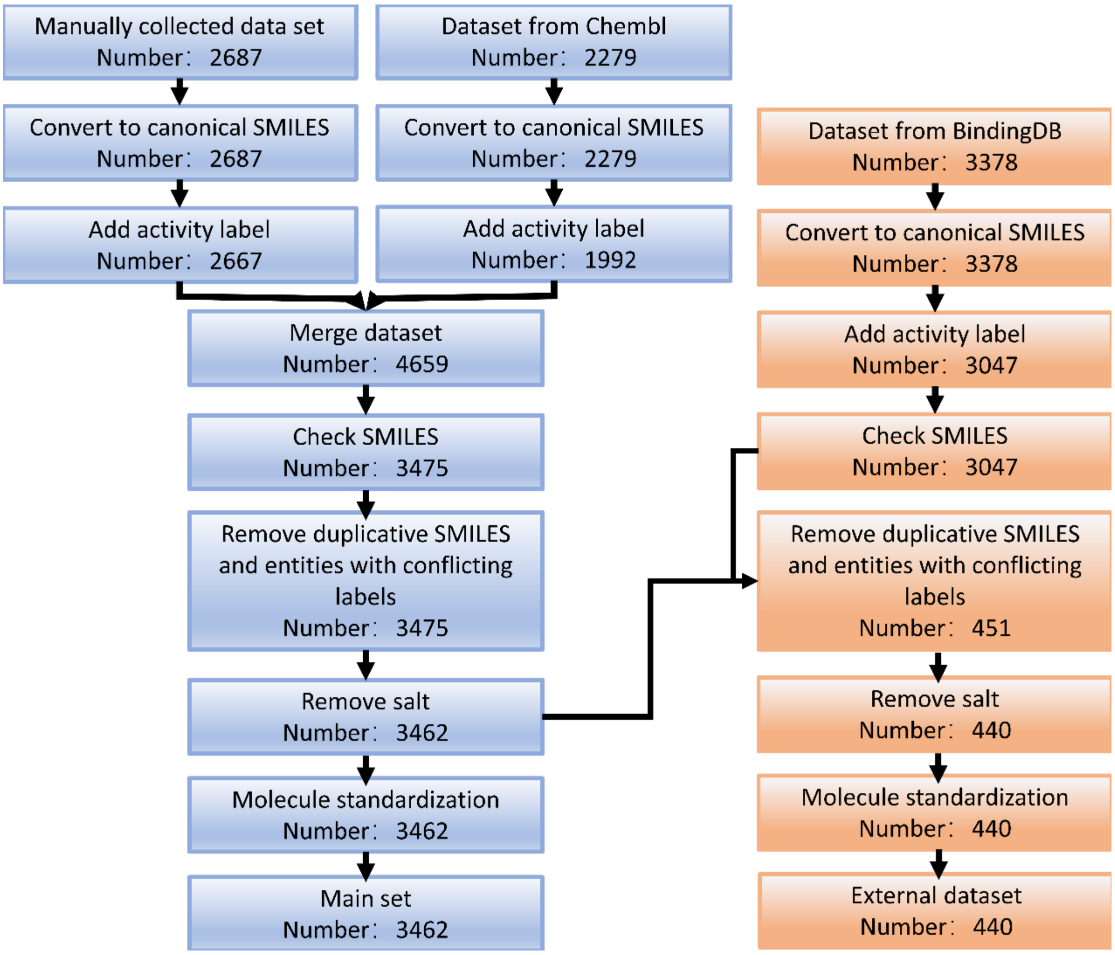

Supplementary Figure 1. The data processing steps

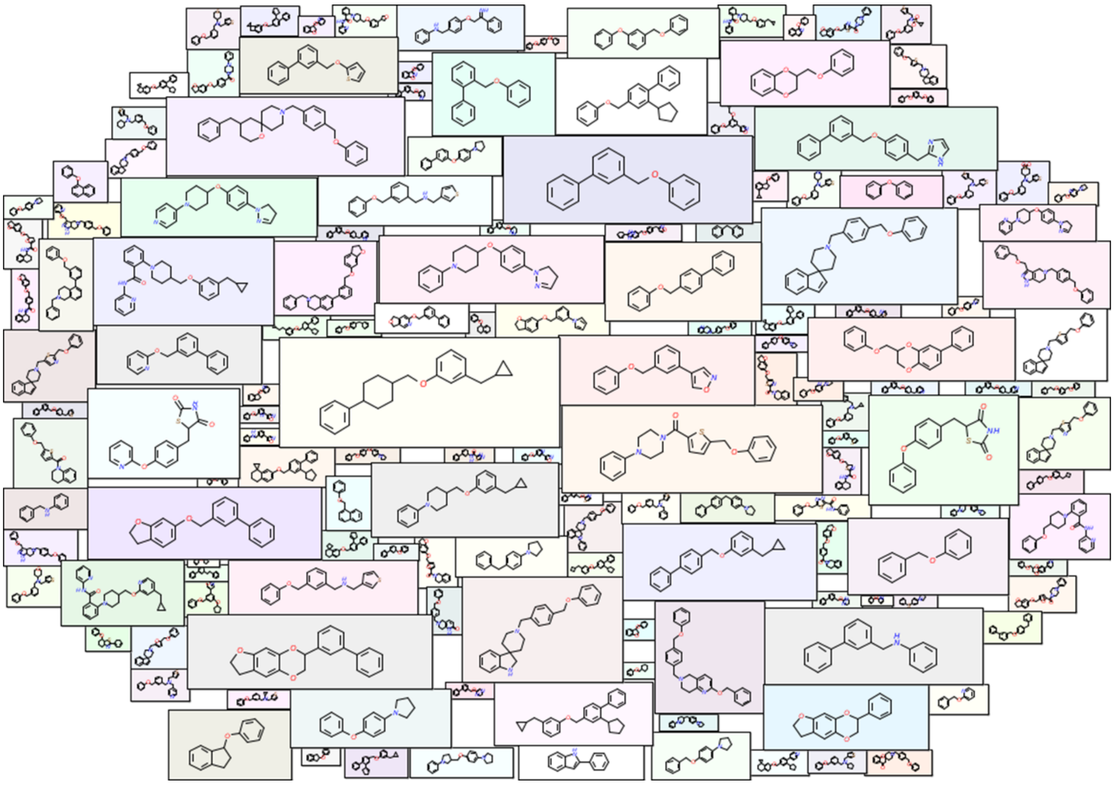

Supplementary Figure 2. The frameworks of GPR40 agonists

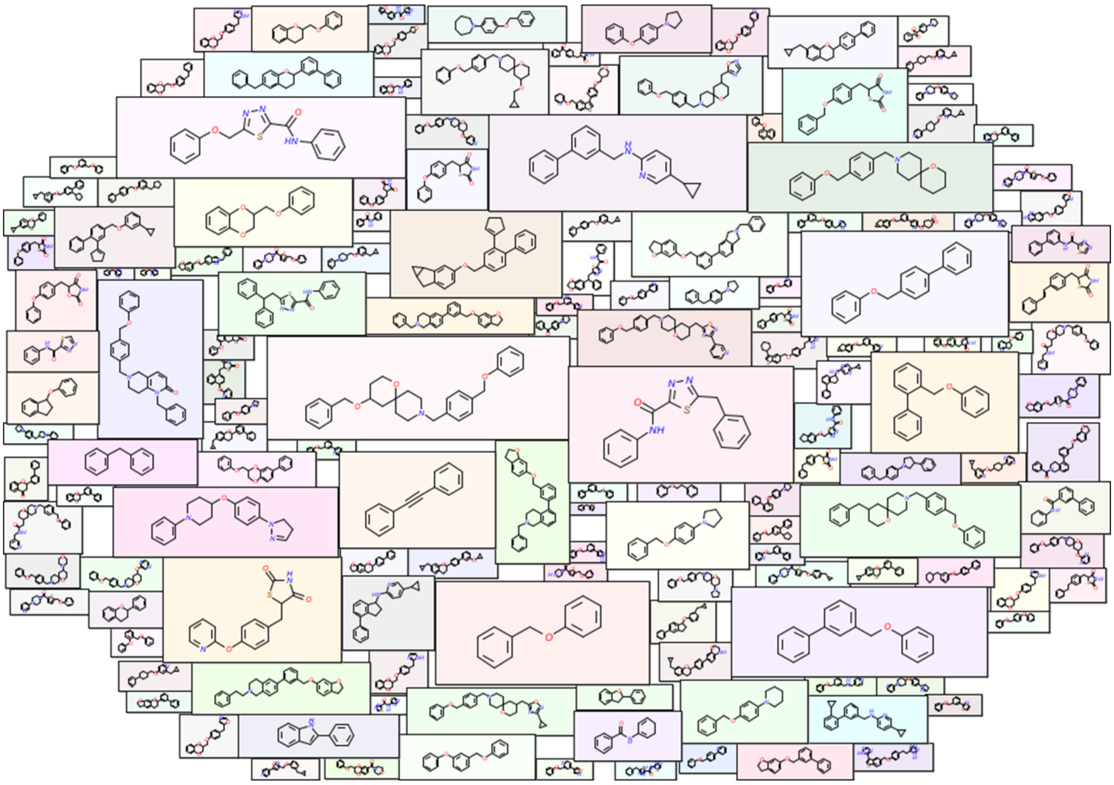

Supplementary Figure 3. The frameworks of GPR40 non-agonists

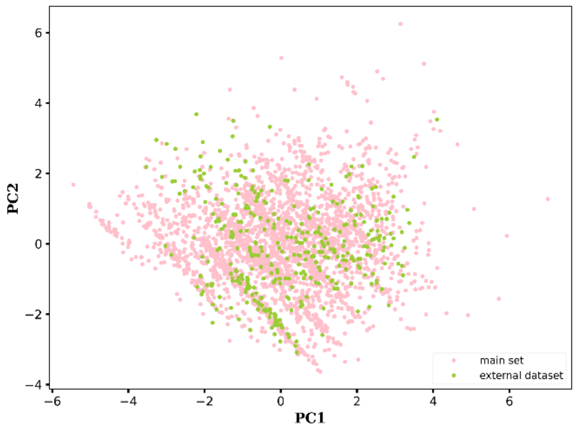

Supplementary Figure 4. The principal component analysis of main set and external dataset based on six molecular properties. The compounds in main set are represented by pink points. The compounds in external validation set are represented by green points.

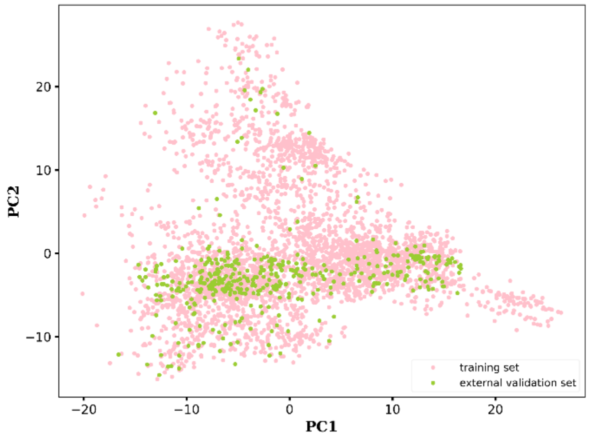

Supplementary Figure 5. The principal component analysis of main set and external dataset using AtomPairFP fingerprint. The compounds in main set are represented by pink points. The compounds in external validation set are represented by green points.

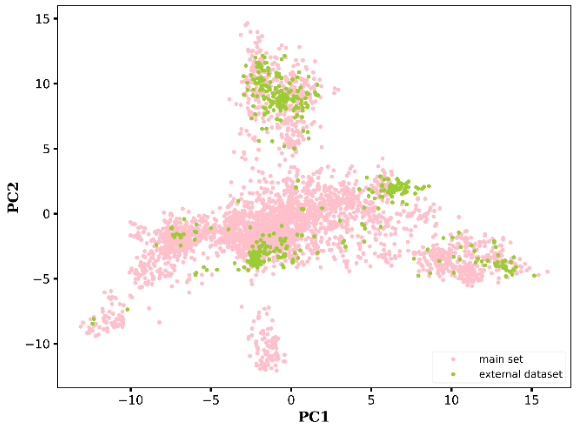

Supplementary Figure 6. The principal component analysis of main set and external dataset using MorganFP fingerprint. The compounds in main set are represented by pink points. The compounds in external validation set are represented by green points.

Supplementary Table 1. The hyperparameters of FCNN, RF and XGBoost models

hyperparameter	FCNN	hyperparameter	RF	hyperparameter	XGBoost
layer_sizes	800	bootstrap	TRUE	max_depth	5
weight_init_stddevs	0.02	class_weight	balanced	learning_rate	0.05
bias_init_consts	1	criterion	gini	n_estimators	1000
dropouts	0.5	max_depth	None	gamma	0
weight_decay_penalty	0.1	max_features	auto	min_child_weight	5
weight_decay_penalty_type	l2	max_leaf_nodes	None	max_delta_step	1
batch_size	8192	min_impurity_decrease	0	subsample	0.53
learning_rate	0.001	min_impurity_split	None	colsample_bytree	0.66
optimizer	Adam	min_sample_leaf	1	cosample_bylevel	1
		min_sample_split	2	reg_alpha	0
		min_weight_fraction_leaf	0	reg_lambda	1
		n_estimators	50	scale_pos_weight	1
		n_jobs	-1	base_score	0.5
		oob_score	False		
		random_state	None		
		verbose	0		
		warm_start	False		

Supplementary Table 2. The hyperparameters of LR and D-MPNN models

hyperparameter	LR	hyperparameter	D-MPNN	hyperparameter	SVM
C	1	activation	ReLU	C	1.0
class_weight	balance	atom_messages	False	kernel	linear
dual	False	batch_size	50	degree	3
fit_intercept	True	bias	False	gamma	auto
intercept_scaling	1	dataset_type	classification	coef0	0.0
max_iter	100	depth	3	shrinking	True
multi_class	ovr	dropout	0	probability	True
n_jobs	-1	ensemble_size	1	tol	1e-3
penalty	l2	epochs	30	cache_size	200
random_state	None	features_scaling	True	class_weight	None
solver	liblinear	ffn_hidden_size	300	verbose	False
tol	0.0001	ffn_num_layers	2	max_iter	-1
verbose	0	final_lr	0.0001	decision_function_shape	ovr
warm_start	False	hidden_size	300	break_ties	False
		init_lr	0.0001	random_state	None
		max_lr	0.001		
		split_sizes	0.8, 0.1, 0.1		
		split_type	random		
		warmup_epochs	2		

Supplementary Table 3. The name, size, and source of the fingerprints and descriptors.

Representation	Size	Ref. (DOI)
MorganFP<br>(Extended-connectivity fingerprint)	204<br>8	10.1021/ci100050t
RDkitFP<br>(Topological Fingerprints)	204<br>8	https://rdkit.org/docs/GettingStartedInPython.html#topologi-<br>cal-fingerprints
AtomPairFP<br>(Atom-pair fingerprint)	204<br>8	https://rdkit.org/docs/GettingStartedInPython.html#atom-<br>pairs-and-topological-torsions
TorsionFP<br>(Topological torsion fingerprint)	204<br>8	https://rdkit.org/docs/GettingStartedInPython.html#atom-<br>pairs-and-topological-torsions
AvalonFP<br>(Avalon fingerprint)	204<br>8	10.1021/ci050413p
EstateFP<br>(Electrotopological state indices for atom types)	79	10.1021/ci00028a014
MACCSFP<br>(Molecular access system fingerprint)	166	10.1021/ci010132r
PharmacoErGFP<br>(Pharmacophore-type extended reduced graph fingerprint)	441	10.1021/ci050457y
PharmacoPFP<br>(Pharmacophore Fingerprint)	330	10.1021/ci980159j
PubChemFP<br>(PubChem fingerprint)	881	list_fingerprints.pdf(ohio-state.edu)
MHFP6<br>(MinHash fingerprint, up to six bonds)	204<br>8	10.1186/s13321-018-0321-8
MAP4<br>(MinHashed atom-pair fingerprint up to a diameter of four bonds)	204<br>8	10.1186/s13321-020-00445-4
MOE<br>(Molecular Operating Environment)	606	10.1186/s13321-018-0258-y
Property<br>(Molecular physiochemical properties)	18	wanxiang.shen@u.nus.edu
Constitution<br>(Molecular constitutional indices based on topological structure)	63	10.1002/cmdc.200900317
Autocorr<br>(Molecular autocorrelation descriptors)	606	10.1186/s13321-018-0258-y
Fragment	85	https://www.rdkit.org/docs/source/rdkit.Chem.Frag-<br>ments.html
Charge<br>(Gasteiger atomic charges)	25	https://github.com/gadsbyfly/PyBioMed
Estate	316	10.1186/s13321-018-0258-y
Connectivity<br>(Molecular connectivity indices based ontopological)	56	10.1186/s13321-018-0258-y
Topology<br>(Topology indexes)	24	https://rdkit.org/docs/index.html
Kappa	8	https://github.com/gadsbyfly/PyBioMed
Path	18	http://rdkit.org/docs/source/rdkit.Chem.rdmolops.html
Matrix<br>(AdjacencyMatrix, BaryszMatrix, DetourMatrix, Distance-Matrix descriptors)	142	10.1186/s13321-018-0258-y
InfoContent<br>(Information Content descriptors)	42	10.1186/s13321-018-0258-y

Supplementary Table 4. The performance comparison between the ensemble model and the top 20 baseline models was evaluated using different metrics on the external dataset.

algorithm	representation	ROC AUC	Accuracy	Cohen_kappa[a]	F1[b]	FN[c]	FP[d]	MCC[e]	NPV[f]	Precision	Recall
Ensemble model		**0.9496**	**0.9045**	**0.6839**	**0.9414**	15.4	26.6	**0.6903**	0.8086	**0.9273**	0.9564
FCNN	AtomPairFP	0.9282	0.8868	0.6156	0.9311	16.6	33.2	0.6220	0.7681	0.9103	0.9530
D-MPNN	Topology	0.9045	0.8955	0.6328	0.9369	11.4	34.6	0.6468	0.8284	0.9083	0.9677
RF	Autocorr	0.9274	0.8705	0.4784	0.9246	**3.6**	53.4	0.5388	**0.9077**	0.8675	**0.9898**
D-MPNN	Kappa	0.8994	0.8827	0.5974	0.9288	16.6	35	0.6049	0.7597	0.9060	0.9530
D-MPNN	Property	0.9099	0.8882	0.6079	0.9323	13.2	36	0.6260	0.8168	0.9048	0.9626
D-MPNN	InfoContent	0.9155	0.8836	0.5718	0.9305	10.4	40.8	0.5988	0.8305	0.8946	0.9705
XGB	AtomPairFP	0.9024	0.8659	0.5786	0.9163	29.4	29.6	0.5804	0.6694	0.9163	0.9167
D-MPNN	Connectivity	0.8909	0.8750	0.5156	0.9264	7	48	0.5614	0.8712	0.8790	0.9802
RF	MorganFP	0.8868	0.8582	0.4484	0.9168	9.4	53	0.4871	0.7870	0.8664	0.9734
D-MPNN	AtomPairFP	0.9019	0.8673	0.5797	0.9172	28.2	30.2	0.5841	0.6832	0.9154	0.9201
RF	AtomPairFP	0.8951	0.8259	0.4186	0.8934	31.8	44.8	0.4209	0.5710	0.8776	0.9099
Logreg	AtomPairFP	0.9050	0.8473	0.5504	0.9025	41.6	25.6	0.5539	0.5981	0.9241	0.8822
D-MPNN	PharmacoErGFP	0.8713	0.8464	0.5498	0.9018	42.6	**25**	0.5541	0.5933	0.9256	0.8793
D-MPNN	Matrix	0.8916	0.8686	0.5309	0.9209	16.6	41.2	0.5520	0.7562	0.8921	0.9530
D-MPNN	N.A.[g]	0.8801	0.8486	0.3581	0.9130	3.8	62.8	0.4359	0.8673	0.8477	0.9892
RF	MAP4	0.8795	0.8268	0.2504	0.9009	6.8	69.4	0.3194	0.7289	0.8331	0.9807
D-MPNN	Path	0.8874	0.8755	0.5210	0.9266	7.2	47.6	0.5623	0.8614	0.8794	0.9796
SVM	AtomPairFP	0.8747	0.8405	0.5103	0.8996	38	32.2	0.5117	0.5948	0.9073	0.8924
FCNN	MAP4	0.8908	0.8427	0.3518	0.9089	8	61.2	0.4036	0.7518	0.8496	0.9773
XGB	MAP4	0.8782	0.8400	0.4687	0.9018	29.4	41	0.4726	0.6154	0.8878	0.9167

Cohen_kappa[a]: Cohen's Kappa; F1[b]: F1 score; FN[c]: False Negative; FP[d]: False Positive; MCC[e]: Matthews Correlation Coefficient; NPV[f]: Negative Predictive Value. N.A.[g]: The D-MPNN was trained without additional molecular representations as input. All models were developed using 5-fold cross-validation method. For evaluations on more metrics (such as F1, MCC, etc) please check github (https://github.com/Jiamin-Yang/ensemble_model) and zenodo (DOI 10.5281/zenodo.7641975)

Supplementary Table 5. The performance comparison between the ensemble model and the top 20 baseline models was evaluated using different metrics on the test set.

algorithm	representation	ROC AUC	Accuracy	Cohen_kappa[a]	F1[b]	FN[c]	FP[d]	MCC[e]	NPV[f]	Precision	Recall
Ensemble model		**0.9524**	0.9101	**0.7421**	0.9419	16.2	**15**	**0.7438**	0.7965	**0.9446**	0.9396
FCNN	AtomPairFP	0.9471	0.9107	0.7312	0.9434	11	20	0.7347	0.8404	0.9285	0.9591
D-MPNN	Topology	0.9462	**0.9112**	0.7347	**0.9436**	11	19.8	0.7373	**0.8417**	0.9288	0.9590
RF	Autocorr	0.9442	0.9095	0.7308	0.9424	12	19.4	0.7330	0.8306	0.9300	0.9553
D-MPNN	Kappa	0.9442	0.8991	0.7054	0.9354	15.6	19.4	0.7081	0.7932	0.9294	0.9421
D-MPNN	Property	0.9442	0.8963	0.6844	0.9345	12.2	23.8	0.6896	0.8175	0.9156	0.9546
D-MPNN	InfoContent	0.9439	0.8980	0.6923	0.9354	12.6	22.8	0.6960	0.8149	0.9186	0.9531
XGB	AtomPairFP	0.9429	0.9061	0.7184	0.9403	12	20.6	0.7215	0.8282	0.9261	0.9553
D-MPNN	Connectivity	0.9426	0.9078	0.7246	0.9414	11.8	20.2	0.7275	0.8324	0.9274	0.9561
RF	MorganFP	0.9423	0.8963	0.6889	0.9341	13.6	22.4	0.6920	0.8045	0.9197	0.9494
D-MPNN	AtomPairFP	0.9420	0.8991	0.6932	0.9364	11.4	23.6	0.6977	0.8263	0.9162	0.9576
RF	AtomPairFP	0.9417	0.9066	0.7190	0.9408	11.4	21	0.7222	0.8335	0.9248	0.9576
Logreg	AtomPairFP	0.9416	0.8997	0.7000	0.9363	13.2	21.6	0.7028	0.8103	0.9224	0.9508
D-MPNN	PharmacoErGFP	0.9407	0.9009	0.6980	0.9375	11	23.4	0.7028	0.8319	0.9170	0.9591
D-MPNN	Matrix	0.9404	0.9032	0.7206	0.9376	16.4	17.2	0.7237	0.7935	0.9369	0.9390
D-MPNN	N.A.[g]	0.9400	0.8963	0.6915	0.9338	14.6	21.4	0.6952	0.7983	0.9231	0.9455
RF	MAP4	0.9398	0.9055	0.7195	0.9397	13.2	19.6	0.7216	0.8160	0.9292	0.9508
D-MPNN	Path	0.9394	0.8968	0.6933	0.9343	14	21.8	0.6957	0.8021	0.9214	0.9478
SVM	AtomPairFP	0.9393	0.8951	0.6824	0.9337	12.6	23.8	0.6871	0.8130	0.9153	0.9531
FCNN	MAP4	0.9389	0.8853	0.6338	0.9289	**9**	30.8	0.6485	**0.8417**	0.8944	**0.9665**
XGB	MAP4	0.9389	0.8974	0.6939	0.9348	13.4	22.2	0.6964	0.8078	0.9201	0.9501

Cohen_kappa[a]: Cohen's Kappa; F1[b]: F1 score; FN[c]: False Negative; FP[d]: False Positive; MCC[e]: Matthews Correlation Coefficient; NPV[f]: Negative Predictive Value. N.A.[g:] The D-MPNN was trained without additional molecular representations as input. All models were developed using 5-fold cross-validation method. For evaluations on more metrics (such as F1, MCC, etc) please check github (https://github.com/Jiamin-Yang/ensemble_model) and zenodo (DOI 10.5281/zenodo.7641975)

Supplementary Table 6. Performance comparison of FCNN models using fingerprints and chiral fingerprints

Dataset	Representation	ROC AUC	Accuracy	Recall	Precision	F1 score	Cohen_kappa[c]	MCC[d]	NPV[e]	FP[f]	FN[g]
Test	AtomPairFP	0.9417	0.9066	0.9576	0.9248	0.9408	0.7190	0.7222	0.8335	21	11.4
	AtomPairFPC[a]	0.9444	0.9066	0.9568	0.9255	0.9408	0.6899	0.6925	0.8028	24.4	14.6
External	AtomPairFP	0.9282	0.8868	0.9530	0.9103	0.9311	0.7026	0.7058	0.8202	23	12.8
	AtomPairFPC[a]	0.9225	0.8936	0.9688	0.9053	0.9359	0.6661	0.6696	0.7936	27.4	15.2
Test	MorganFP	0.9315	0.9009	0.9643	0.9130	0.9378	0.6929	0.7058	0.8464	24.8	9.6
	MorganFPC[b]	0.9352	0.8976	0.9591	0.9136	0.9357	0.5365	0.6696	0.8139	41.4	8
External	MorganFP	0.8400	0.8232	0.9819	0.8292	0.8991	0.5903	0.6106	0.8131	34.8	9.2
	MorganFPC[b]	0.8301	0.8218	0.9807	0.8287	0.8983	0.4257	0.4748	0.7980	52.6	6.8

AtomPairFPC[a]: chiral AtomPairFP. MorganFPC[b]: chiral MorganFP. Cohen_kappa[c]：Cohen's Kappa. MCC[d]：Matthews Correlation Coefficient. NPV[e]：Negative Predictive Value. FP[f]：False Positive. FN[g]；False Negative. All models were developed using 5-fold cross-validation method.

Supplementary Table 7. Performance evaluation of FCNN models with different optimizers and hidden node numbers

optimizer	no. of the nodes[a]	ROC AUC		Accuracy	
		Test[b]	External[b]	Test[b]	External[b]
adam	200	0.9391±0.0233	0.9241±0.0112	0.9063±0.0113	0.8930±0.0097
	400	0.9388±0.0238	0.9235±0.0104	0.9043±0.0110	0.8845±0.0125
	600	0.9397±0.0248	0.9271±0.0115	0.9084±0.0131	**0.8936**±0.0130
	800	0.9408±0.0228	**0.9289**±0.0108	0.9078±0.0113	0.8934±0.0112
	1000	0.9417±0.0224	0.9271±0.0123	**0.9098**±0.0127	0.8895±0.0143
rmsprop	200	0.9393±0.0241	0.9243±0.0079	0.9035±0.0135	0.8850±0.0119
	400	0.9414±0.0235	0.9267±0.0067	0.8994±0.0220	0.8705±0.0350
	600	0.9389±0.0245	0.9259±0.0149	0.8939±0.0157	0.8684±0.0259
	800	**0.9419**±0.0231	0.9247±0.0104	0.8963±0.0265	0.8705±0.0293
	1000	0.9409±0.0233	0.9222±0.0136	0.8859±0.0310	0.8507±0.0363

no. of nodes[a]: the nodes in the hidden layer of FCNN. Test[b] or External[b]: evaluating the ensemble model based on a test set or external dataset. All models were developed using 5-fold cross-validation method.

Supplementary Table 8. Assessment of FCNN model performance with various optimizers and hidden layer sizes

no. of nodes in hidden layer 1[a]	no. of nodes in hidden layer 2[a]	ROC AUC		Accuracy	
		Test[b]	External[b]	Test[b]	External[b]
30	30	0.9324±0.0240	0.9126±0.0079	0.9037±0.0135	0.8850±0.0090
40	40	0.9328±0.0254	0.9160±0.0099	0.9037±0.0151	0.8864±0.0059
50	50	0.9346±0.0283	0.9100±0.0088	0.9026±0.0158	0.8827±0.0138
50	100	0.9327±0.0264	0.9116±0.0113	0.9043±0.0111	0.8895±0.0163
50	200	0.9316±0.0281	**0.9199**±0.0131	0.9037±0.0139	0.8850±0.0089
50	400	0.9300±0.0267	0.9157±0.0125	0.9020±0.0115	0.8827±0.0095
100	100	0.9358±0.0258	0.9187±0.0087	**0.9072**±0.0127	**0.8936**±0.0123
100	200	**0.9363**±0.0247	0.9174±0.0124	0.9026±0.0147	0.8800±0.0137

no. of nodes in hidden layer 1[a], no. of nodes in hidden layer 2[a]: the nodes in the hidden layer of FCNN. Test[b] or External[b]: evaluating the ensemble model based on a test set or external dataset. All models were developed using 5-fold cross-validation method.

Paragraph 1

Most of the compound data points were gathered from calcium flux analysis or IP1 accumulation measurements on CHO cells or HEK-293 cells. Other methods for determining activity include the aequorin assay on CHO cells, Beta-arrestin 2 recruitment on HEK- 293 cells, and so on. These assays were conducted under different conditions, such as varying concentrations of human serum and measurements taken at different intervals. Additionally, the CHO cell line refers to a Chinese hamster ovary cell line, and HEK 293 cells are a type of human embryonic kidney cell line.
